# Implementing EM and Viterbi algorithms for Hidden Markov Model in linear memory

**DOI:** 10.1186/1471-2105-9-224

**Published:** 2008-04-30

**Authors:** Alexander Churbanov, Stephen Winters-Hilt

**Affiliations:** 1 The Research Institute for Children, 200 Henry Clay Ave., New Orleans, LA 70118, USA; 2 Department of Computer Science, University of New Orleans, New Orleans, LA, 70148, USA

## Abstract

**Background:**

The Baum-Welch learning procedure for Hidden Markov Models (HMMs) provides a powerful tool for tailoring HMM topologies to data for use in knowledge discovery and clustering. A linear memory procedure recently proposed by *Miklós, I. and Meyer, I.M. *describes a memory sparse version of the Baum-Welch algorithm with modifications to the original probabilistic table topologies to make memory use independent of sequence length (and linearly dependent on state number). The original description of the technique has some errors that we amend. We then compare the corrected implementation on a variety of data sets with conventional and checkpointing implementations.

**Results:**

We provide a correct recurrence relation for the emission parameter estimate and extend it to parameter estimates of the Normal distribution. To accelerate estimation of the prior state probabilities, and decrease memory use, we reverse the originally proposed forward sweep. We describe different scaling strategies necessary in all real implementations of the algorithm to prevent underflow. In this paper we also describe our approach to a linear memory implementation of the Viterbi decoding algorithm (with linearity in the sequence length, while memory use is approximately independent of state number). We demonstrate the use of the linear memory implementation on an extended Duration Hidden Markov Model (DHMM) and on an HMM with a spike detection topology. Comparing the various implementations of the Baum-Welch procedure we find that the checkpointing algorithm produces the best overall tradeoff between memory use and speed. In cases where sequence length is very large (for Baum-Welch), or state number is very large (for Viterbi), the linear memory methods outlined may offer some utility.

**Conclusion:**

Our performance-optimized Java implementations of Baum-Welch algorithm are available at . The described method and implementations will aid sequence alignment, gene structure prediction, HMM profile training, nanopore ionic flow blockades analysis and many other domains that require efficient HMM training with EM.

## Background

Hidden Markov Models (HMMs) are a widely accepted modeling tool [1] used in various domains, such as speech recognition [2] and bioinformatics [3]. An HMM can be described as a stochastic finite state machine where each transition between hidden states ends with a symbol emission. The HMM can be represented as a directed graph with *N *states where each state can emit either a discrete character or a continuous value drawn from a Probability Density Function (PDF).

We are interested in a distributed HMM analysis of the channel current blockade signal caused by a single DNA hairpin molecule held in a nanopore detector [4,5]. The molecules examined frequently produce toggles with stationary statistical profiles for thousands of milliseconds. With a sampling rate of 20 *μ*s, processing even a modest blockade signal of 200 ms duration (10,000 sample points) becomes problematic, mostly because of the size of the dynamic programming tables required in the conventional implementations of the HMM's Baum-Welch and Viterbi decoding algorithms. Since we are also trying to model durations [6] and spike phenomena [7], by increasing the number of HMM states, conventional HMM implementations are found to be prohibitively expensive in terms of memory use.

The Baum-Welch algorithm is an Expectation Maximization (EM) algorithm invented by Leonard E. Baum and Lloyd R. Welch, and first appears in [8]. A later refinement, Hirschberg's algorithm for an HMM [9], reduces the memory footprint by recursively halving the *pairwise alignment *dynamic programming table for sequences of comparable size. In our application domain, the length of the observed emission sequence (in the case of nanopore ionic flow blockade analysis or gene structure prediction) is prohibitively long compared to the number of HMM states. Further, Baum-Welch requires multiple paths, instead of the most likely one, making this strategy less than optimal.

The checkpointing algorithm [10-12] implements the Baum-Welch algorithm in $O(\sqrt{T}N)$ memory and in *O*(*TNQ*_*max *_+ *T*(*Q *+ *E*)) processor time, where *T *is the length of the observed sequence, *Q*_*max *_is the maximum HMM node out-degree, *E *is the number of free emission parameters and *Q *is the number of free transition parameters. It divides the input sequence into $\sqrt{T}$ blocks of $\sqrt{T}$ symbols each, and, during the forward pass, retains the first column of the forward probability table for each block. When the reverse sweep starts, the forward values for each block are sequentially re-evaluated, beginning with their corresponding checkpoints, to update the parameter estimates.

Further refinement to the algorithm, as described in [13] and amended here, has rendered the memory demands independent of the observed sequence length, with *O*(*N*(*Q *+ *ED*)) memory usage and *O*(*TNQ*_*max*_(*Q *+ *ED*)) running time, where *D *is the dimensionality of a vector that stores statistics on the emission PDF parameter estimates. Performance of the various algorithms is summarized in Table 1. In this work, we also present a modification of one of the key HMM algorithms, the Viterbi algorithm, improving the memory profile without affecting the execution time.

**Table 1:** The computational expense of different algorithm implementations running on HMM.

Algorith	Canonical		Checkpointing		Linear	
			
Viterbi	Time	*O*(*TNQ*_*max*_)	Time	*O*(*TNQ*_*max*_)	Time	*O*(*TNQ*_*max*_)
	Space	*O*(*TN*)	Space	$O(\sqrt{T}N+T)$	Space	*O*(*T*)
Baum-Welch	Time	*O*(*TNQ*_*max *_+ *T *(*Q *+ *E*))	Time	*O*(*TNQ*_*max *_+ *T *(*Q *+ *E*))	Time	*O*(*TNQ*_*max*_(*Q *+ *ED*))
	Space	*O*(*TN*)	Space	$O(\sqrt{T}N)$	Space	*O*(*N*(*Q *+ *ED*))

## Methods and Results

### HMM definition, EM learning and Viterbi decoding

The following parameters describe the conventional HMM implementation according to [14]:

• A set of states *S *= {*S*_1_,..., *S*_*N*_} with *q*_*t *_being the state visited at time *t*,

• A set of PDFs *B *= {*b*_1_(*o*),..., *b*_*N*_(*o*)}, describing the emission probabilities *b*_*j*_(*o*_*t*_) = *p*(*o*_*t*_|*q*_*t *_= *S*_*j*_) for 1 ≤ *j *≤ *N*, where *o*_*t *_is the observation at time-point *t *from the sequence of observations *O *= {*o*_1_,..., *o*_*T*_},

• The state-transition probability matrix *A *= {*a*_*i*, *j*_} for 1 ≤ *i*, *j *≤ *N*, where *a*_*i*, *j *_= *p*(*q*_*t *+ 1 _= *S*_*j*_|*q*_*t *_= *S*_*i*_),

• The initial state distribution vector *Π *= {*π*_1_,..., *π*_*N*_}.

A set of parameters *λ *= (*Π*, *A*, *B*) completely specifies an HMM. Here we describe the HMM parameter update rules for the EM learning algorithm rigorously derived in [15]. The Viterbi algorithm, as shown in Table 2, is a dynamic programming algorithm that runs an HMM to find the most likely sequence of hidden states, called the Viterbi path, that result in an observed sequence. When training the HMM using the Baum-Welch algorithm (an Expectation Maximization procedure), first we need to find the expected probabilities of being at a certain state at a certain time-point using the forward-backward procedure as shown in Table 2. The forward, backward, and Viterbi algorithms take *O*(*TNQ*_*max*_) time to execute.

**Table 2:** The Viterbi decoding, forward and backward procedures.

Forward procedure	Backward procedure	Viterbi algorithm
*α*_*t*_(*i*) ≡ *p *(*o*_1_,..., *o*_*t*_|*q*_*t *_= *S*_*i*_, *λ*)	*β*_*t*_(*i*) ≡ *p *(*o*_*t *+ 1_,..., *o*_*T*_|*q*_*t *_= *S*_*i*_, *λ)*	• Initially *δ*_1_(*i*) = *π*_*i*_*b*_*i*_(*o*_1_), *ψ*_1_(*i*) = 0 for 1 ≤ *i *≤ *N*,
• Initially *α*_1_(*i*) = *π*_*i*_*b*_*i*_(*o*_1_) for 1 ≤ *i *≤ *N*,	• Initially *β*_*T*_(*i*) = 1 for 1 ≤ *i *≤ *N*,	• $\begin{array}{l} \delta_{t}(j)=\underset{1\leq i\leq N}{\max}[\delta_{t-1}(i)a_{i,j}]b_{j}(o_{t}),\\ \psi_{t}(j)=\underset{1\leq i\leq N}{\arg\max}[\delta_{t-1}(i)a_{i,j}] \end{array}$ for *t *= 2,..., *T *and 1 ≤ *j *≤ *N*,
• $\alpha_{t}(j)=\left[\sum_{i=1}^{N}\alpha_{t-1}(i)a_{i,j}\right]b_{j}(o_{t})$ for *t *= 2, 3,..., *T *and 1 ≤ *j *≤ *N*,	• $\beta_{t}(i)=\sum_{j=1}^{N}a_{i,j}b_{j}(o_{t+1})\beta_{t+1}(j)$ for *t *= *T *- 1,..., 1 and 1 ≤ *i *≤ *N*,	• Finally $q_{T}^{*}=\underset{1\leq i\leq N}{\arg\max}[\delta_{T}(i)],q_{t}^{*}=\psi_{t+1}(q_{t+1}^{*})$ for *t *= *T *- 1,..., 1 with optimal path $Q^{*}=\{q_{1}^{*},...,q_{T}^{*}\}$.
• Finally $p(O|\lambda )=\sum_{i=1}^{N}\alpha_{T}(i)$ is the sequence *likelihood*	• Finally $p(O|\lambda )=\sum_{i=1}^{N}\pi_{i}b_{i}(o_{1})\beta_{1}(i)$.	

Let us define *ξ*_*t*_(*i*, *j*) as the probability of being in state *i *at time *t*, and state *j *at time *t *+ 1, given the model and the observation sequence

(1) $$\xi_{t}(i,j)=p(q_{t}=S_{i},q_{t+1}=S_{j}|O,\lambda )=\frac{\alpha_{t}(i)a_{i,j}b_{j}(o_{t+1})\beta_{t+1}(j)}{p(O|\lambda )},$$

and *γ*_*t*_(*i*) as the probability of being in state *i *at time *t*, given the observation sequence and the model

(2) $$\gamma_{t}(i)=p(q_{t}=S_{i}|O,\lambda )=\frac{\alpha_{t}(i)\beta_{t}(i)}{\sum_{i=1}^{N}\alpha_{t}(i)\beta_{t}(i)}=\sum_{j=1}^{N}\xi_{t}(i,j).$$

The HMM maximization step using these probabilities is shown in Table 3. The conventional EM procedure for HMM [14] takes *O*(*TN*) memory and *O*(*TNQ*_*max *_+ *T *(*Q *+ *E*)) processor time. An HMM containing empty internal states (see for example [3]) and Hierarchical HMM (HHMM) could be converted into canonical HMM form through stack transformation as discussed in [16].

**Table 3:** The maximization step in HMM learning. states.

Initial probability estimate	Transition probability estimate	Emission parameters estimate
${\overset{˄}{\pi }}_{i}$ = *γ*_1_(*i*), for 1 ≤ *i *≤ *N*.	• Gaussian emission ${\hat{a}}_{i,j}=\frac{\sum_{t=1}^{T-1}\xi_{t}(i,j)}{\sum_{t=1}^{T-1}\gamma_{t}(i)}$, for 1 ≤ *i, j *≤ *N*.	$\begin{array}{l} {\hat{b}}_{j}(o)\to \mu =\frac{\sum_{t=1}^{T}o_{t}\gamma_{t}(j)}{\sum_{t=1}^{T}\gamma_{t}(j)},\\ {\hat{b}}_{j}(o)\to \sigma^{2}=\frac{\sum_{t=1}^{T}{\left(o_{t}-{\hat{\mu }}_{j}\right)}^{2}\gamma_{t}(j)}{\sum_{t=1}^{T}\gamma_{t}(j)} \end{array}$, for 1 ≤ *j *≤ *N*,
		• Discrete emission ${\hat{b}}_{j}(k)=\frac{\sum_{t=1}^{T}\delta (o_{t}=v_{k})\gamma_{t}(j)}{\sum_{t=1}^{T}\gamma_{t}(j)}$, for 1 ≤ *j *≤ *N*. and 1 ≤ *k *≤ *K*, where *v*_1_,..., *v*_*K *_is the set of possible dircrete observations.

### Forward sweep strategy explained

**Figure 1:**
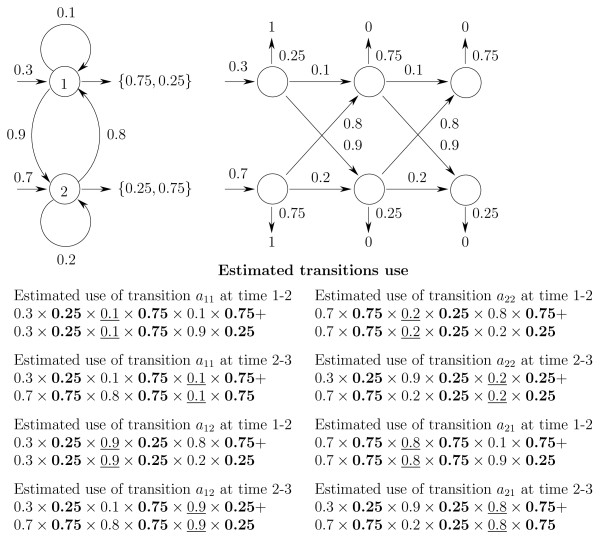
**Time trellis for simple model where possible emissions of 0 and 1 are shown above and below trellis.** Probabilities of emissions that happen after each transition are shown in bold and transitions of interest taken at certain time-point are underlined.

Figure 1 outlines initial, simple transition probability calculations for all possible paths through a "toy" HMM. In Figure 1, to estimate the probability of transition from state 1 to state 2 (1 → 2), we calculate the probability of transition utilization at time intervals 1–2 and 2–3 as:

*p*(Making transition 1 → 2 at time 1–2) = *α*_1_(1) × *a*_1,2 _× *b*_2_(*o*_2_) × *β*_2_(2), *p*(Making transition 1 → 2 at time 2–3) = *α*_2_(1) × *a*_1,2 _× *b*_2_(*o*_3_) × *β*_3_(2).

In particular, to estimate the probability of transition 1 → 2 at time interval 1–2 we calculate the sum of probabilities of all possible paths that lead to state 1 at time-point 1 (forward probability *α*_1_(1)). Then we multiply this probability of being in state 1 by the transition *a*_1,2 _and emission *b*_2_(*o*_2_) probabilities.

Further multiplication by the sum of probabilities of all possible paths from state 2 at time 2 until the end of the emission sequence (backward probability *β*_2_(2)), is the expected probability of transition use. The sum of these estimates at time-points 1–2 and 2–3 is equivalent to the transition probability estimate in Table 3 (prior to normalization).

According to [13]* t*_*i*, *j*_(*t*, *m*) is the weighted sum of probabilities of all possible state paths that emit subsequence *o*_1_,..., *o*_*t *_and finish in state *m*, taking an *i *→ *j *transition at least once where the weight of each state path is the number of *i *→ *j *transitions taken. Processing of the entire *t*_*i*, *j*_(*t*, *m*) recurrence takes memory proportional to *O*(*NQ*) and processor time *O*(*TNQQ*_*max*_). Initially we have *t*_*i*, *j*_(1, *m*) = 0 since no transitions have been made. After initialization, we perform the following recurrence operations:

(3) $$t_{i,j}(t,m)=\alpha_{t-1}(i)a_{i,m}b_{m}(o_{t})\delta (m=j)$$

(4) $$+\sum_{n=1}^{N}t_{i,j}(t-1,n)a_{n,m}b_{m}(o_{t}),$$

where $\delta (m=j)=\{\begin{array}{ll} 1, & \begin{array}{cc} \text{if} & m=j \end{array}\\ 0, & \text{otherwise} \end{array}$. Following equation (1), at a certain time-point *t *we need to score the evidence supporting transition between nodes *i *and *j*, which is the sum of probabilities of all possible state paths that emit subsequence *o*_1_,..., *o*_*t*-1 _and finish in state *i *(forward probability *α*_*t*-1_(*i*)), multiplied by transition *a*_*i*, *j *_and emission *b*_*j*_(*o*_*t*_) probabilities upon arrival to *o*_*t*_. We extend weighted paths containing evidence of *i *→ *j *transitions made at previous time-points 1,..., *t *- 1 further down the trellis in subequation (4). Finally, by the end of the recurrence we marginalize the final state *m *out of probability *t*_*i*, *j*_(*T*, *m*) to get a weighted sum of state paths taking transition *i *→ *j *at various time-points that is equivalent to the numerator in the transition probability estimate shown in Table 3. Thus, we estimate transition utilization using:

$$\begin{array}{cc} t_{i,j}^{END}=\sum_{m=1}^{N}t_{i,j}(T,m), & a_{i,j}=\frac{t_{i,j}^{END}}{\sum_{j\in out(S_{i})}t_{i,j}^{END}}, \end{array}$$

where *out*(*S*_*i*_) is the set of nodes connected by edges from *S*_*i*_.

According to [13]* e*_*i*_(*γ*, *t*, *m*) is the weighted sum of probabilities of all possible state paths that emit subsequence *o*_1_,..., *o*_*t *_and finish in state *m*, for which state *i *emits observation *γ *at least once where the weight of each state path is the number of *γ *emissions that it makes from state *i*.

The following algorithm updates parameters for the set of discrete symbol probability distributions.

Initialization step *e*_*i*_(*γ*, 1, *m*) = *α*_1_(*m*) *δ *(*i *= *m*) *δ *(*γ *= *o*_1_). After initialization we make the recurrence steps, where we correct the emission recurrence presented in [13] [see Additional File 1]:

(5) $$e_{i}(\gamma ,t,m)=\alpha_{t}(m)\delta (i=m)\delta (\gamma =o_{t})$$

(6) $$=\sum_{n=1}^{N}e_{i}(\gamma ,t-1,n)a_{n,m}b_{m}(o_{t}).$$

Finally, by the end of the recurrence we marginalize the final state *m *out of *e*_*i*_(*γ*, *T*, *m*) and estimate the emission parameters through normalization

$$\begin{array}{cc} e_{i}^{END}(\gamma )=\sum_{m=1}^{N}e_{i}(\gamma ,T,m), & {\hat{b}}_{j}(\gamma )=\frac{e_{j}^{END}(\gamma )}{\sum_{\gamma =1}^{D}e_{j}^{END}(\gamma )}. \end{array}$$

The algorithm for discrete emission parameters estimate *B *= {*b*_1_(*o*),..., *b*_*N *_(*o*)*} *takes in *O*(*NED*) memory and *O*(*TNEDQ*_*max*_) time. As discussed [see Subsection *HMM definition, EM learning and Viterbi decoding*] the forward sweep takes *O*(*TNQ*_*max*_) time, where only the values of *α*_*t*-1_(*i*) for 1 ≤ *i *≤ *N *are needed to evaluate *αt*(*i*), thus reducing the memory requirement to *O*(*N*) for the forward algorithm. Computing *e*_*i*_(*γ*, *t*, *m*) takes *O*(*NED*) previous probabilities of *e*_*i*_(*γ*, *t *- 1, *m*) for 1 ≤ *m *≤ *N*, 1 ≤ *i *≤ *E*, 1≤ *γ *≤ *D*. Recurrent updating of each *e*_*i*_(*γ*, *t*, *m*) probability elements takes *O*(*Q*_*max*_) summations, totalling *O*(*TNEDQ*_*max*_).

**Theorem 1 ***e*_*i*_(*γ*, *t*, *m*) *is the weighted sum of probabilities of all possible state paths that emit subsequence o*_1_,..., *o*_*t *_*and finish in state m, for which state i emits observation γ at least once*.

### Proof

**Initialization **The only chance for a path at a time-point 1 to emit symbol *γ *at least once from state *i *and finish in state *m *is *α*_1_(*m*) in case *i *= *m *and *γ *= *o*_1_. Therefore, initialization conditions satisfy definition of *e*_*i*_(*γ*, *t*, *m*).

**Induction **Suppose *e*_*i*_(*γ*, *t *- 1, *m*) represents correct weighted sum of probabilities of all possible state paths that emit subsequence *o*_1_,..., *o*_*t*-1 _and finish in state *m*, for which state *i *emits observation *γ *at least once. We need to prove the above holds for time-point *t*. Following equation (1) in recurrence part (5) we score the evidence of symbol *o*_*t *_emission from state *i *at time-point *t*, which will be further propagated down the trellis in recurrence part (6). Chances of such event is *α*_*t*_(*m*), i.e. sum of probabilities of all possible state paths finishing in state *m *at time-point *t *under conditions *i *= *m *and *γ *= *o*_*t*_. The second part of the recurrence (6) extends the weighted paths containing evidence of *γ *symbol emission from state *i *at previous time-points 1,..., *t *- 1 and finishing in state *n *further down the trellis through available transitions *a*_*n*, *m*_. Thus the definition of *e*_*i*_(*γ*, *t*, *m*) holds true for the time-point *t*.

At the end of recurrence we marginalize the final state *m *out of probability *e*_*i*_(*γ*, *T*, *m*) to get the weighted sum of all state paths making observation *γ *in state *i *at various time-points equivalent to the numerator of the discrete emission parameter estimate in Table 3, which is a weighted sum of all possible paths that score emissions evidence at certain time-points. By normalizing these scores we estimate the emission parameters.

The forward sweep strategy was originally formulated in [13] for HMMs with silent Start/End states, and automatically handles the prior probabilities estimates for the states as standard transitions connecting *Start *with other non-silent states. The prior transition estimates *a*_*Start*, *i *_are naturally involved within recurrent updates of *t*_*i*, *j*_(*t, m*), which takes an additional *O*(*N*^2^) memory if all *N *non-silent states have non-zero priors with time cost *O*(*TN*^2^*Q*_*max*_). In order to compute the prior estimates in the conventional HMM formulation we need to know the backward probability at time-point 1, which requires calculation of the entire backward table. Therefore, in the next section we present a linear memory Baum-Welch algorithm modification built around a backward sweep with scaling, which only involves calculation of *α*_1_(*i*) for 1 ≤ *i *≤ *N *to estimate priors in *O*(*N*) time and *O*(*N*) memory.

### Linear memory Baum-Welch using a backward sweep with scaling

The objective of the algorithm presented in this section is equivalent to that discussed previously [see Section *Forward sweep strategy explained*] based on forward probabilities of state occupation. However, by using the backward probabilities of state occupation we are able to estimate initial HMM state probabilities much more quickly. In the description that follows we introduce a new set of probabilities:

*E*_*i*_(*γ*, *t*, *m*) – the weighted sum of probabilities of all possible state paths that emit subsequence *o*_*t*_,..., *o*_*T *_and finish in state *m*, for which state *i *emits observation *γ *at least once, where the weight of each state path is the number of *γ *emissions that it makes from state *i*.

*T*_*i*, *j*_(*t*, *m*) – the weighted sum of probabilities of all possible state paths that emit subsequence *o*_*t*_,..., *o*_*T *_and finish in state *m*, taking *i *→ *j *transition at least once, where the weight of each state path is the number of *i *→ *j *transitions that it takes.

All calculations are based on backward probability *β*_*t*_(*i*), but there is inevitably insufficient precision to directly represent these values for significantly long emission sequences. Therefore we scale the backward probability during the recursion.

**Figure 2:**
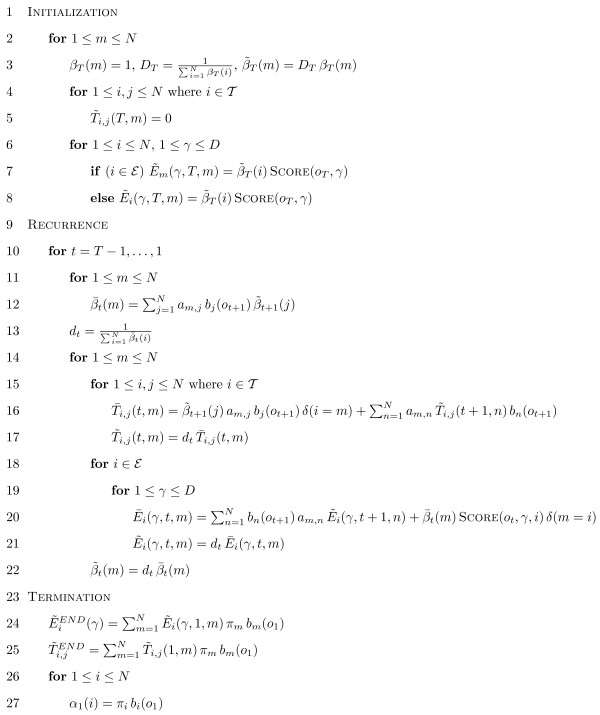
**The linear memory implementation of Baum-Welch learning algorithm for HMM.** This algorithm takes set of HMM parameters *λ *and sequence of symbols *O*. Expected HMM parameters are calculated according to formulas [see Subsection *Parameters update*].

The linear memory Baum-Welch implementation is shown in Figure 2, where E is a set of nodes with free emission parameters and T is a set of nodes with free emanating transitions. Scaling relationships used at every iteration are rigorously proven [see *Appendix A*]. An alternative to scaling is relation (7) presented in [17] showing how to efficiently add log probabilities

(7) $$\log\left(\sum_{t=0}^{N-1}p_{i}\right)=\log p_{0}+\log\left(1+\sum_{i=1}^{N-1}e^{\log p_{i}-\log p_{0}}\right).$$

The scoring functions used for the emissions updates are shown in Table 4. With discrete emission we sum all the backward probabilities of state occupation given discrete symbol emission at certain time-points and later we normalize these counts in (8). In the case of a normally distributed continuous PDF we sum emissions and emission deviation from state *i *mean squared. These sums are scaled by probability of state occupation. We use these counts to estimate the emission mean (9) and variance (10) for a given state.

**Table 4:** The scoring functions for discrete and continuous emissions.

Discrete emission	Continuous Gaussian emission
Score (*o*_*t*_, *γ*, *i*)	Score (*o*_*t*_, *γ*, *i*)
**return ***δ*(*o*_*t *_= *γ*)	**if **(*γ *= 1) **return ***o*_*t*_,
	**if **(*γ *= 2) **return **[*o*_*t *_- (*b*_*i*_(*o*) → *μ*)]^2^,
	**if **(*γ *= 3) **return **1.

#### Parameters update

We estimate the initial probability according to equations presented in Table 3, where *D*_1 _is defined in Appendix A

$${\hat{\pi }}_{i}=\frac{\alpha_{1}(i){\tilde{\beta }}_{i}(1)}{\sum_{i=1}^{N}\alpha_{1}(i){\tilde{\beta }}_{i}(1)}=\frac{\alpha_{1}(i)D_{1}\beta_{1}(i)}{\sum_{i=1}^{N}\alpha_{1}(i)D_{1}\beta_{1}(i)}=\frac{\alpha_{1}(i)\beta_{1}(i)}{\sum_{i=1}^{N}\alpha_{1}(i)\beta_{1}(i)}.$$

The emissions estimate for the discrete case are

(8) $${\hat{b}}_{j}(\gamma )=\frac{{\tilde{E}}_{j}^{END}(\gamma )}{\sum_{\gamma =1}^{D}{\tilde{E}}_{j}^{END}(\gamma )}=\frac{D_{1}E_{j}^{END}(\gamma )}{D_{1}\sum_{\gamma =1}^{D}E_{j}^{END}(\gamma )}=\frac{E_{j}^{END}(\gamma )}{\sum_{\gamma =1}^{D}E_{j}^{END}(\gamma )}.$$

For normally distributed continuous observation PDF

(9) $${\hat{b}}_{j}(o)\to \mu =\frac{{\tilde{E}}_{j}^{END}(1)}{{\tilde{E}}_{j}^{END}(3)}=\frac{D_{1}E_{j}^{END}(1)}{D_{1}E_{j}^{END}(3)}=\frac{E_{j}^{END}(1)}{E_{j}^{END}(3)},$$

(10) $${\hat{b}}_{j}(o)\to \sigma^{2}=\frac{{\tilde{E}}_{j}^{END}(2)}{{\tilde{E}}_{j}^{END}(3)}=\frac{D_{1}E_{j}^{END}(2)}{D_{1}E_{j}^{END}(3)}=\frac{E_{j}^{END}(2)}{E_{j}^{END}(3)}.$$

The transition estimate is calculated as following

$$a_{i,j}=\frac{{\tilde{T}}_{i,j}^{END}}{\sum_{j\in out(S_{i})}{\tilde{T}}_{i,j}^{END}}=\frac{D_{1}T_{i,j}^{END}}{D_{1}\sum_{j\in out(S_{i})}T_{i,j}^{END}}=\frac{T_{i,j}^{END}}{\sum_{j\in out(S_{i})}T_{i,j}^{END}}\text{ for }i\in T.$$

#### Viterbi decoding in linear memory

In this section we describe results when using a "linear memory" modification of the original Viterbi algorithm that was introduced in [18] by Andrew J. Viterbi. As mentioned previously, the Viterbi algorithm is a dynamic programming algorithm for finding the most likely sequence of hidden states, called the "Viterbi path", corresponding to the sequence of observed events in the context of an HMM. Viterbi checkpointing implementation introduced in [11] divides input sequence into $\sqrt{T}$ blocks of $\sqrt{T}$ symbols each and during the first Viterbi pass keeps only the first column of the *d *table for each block. The reconstruction of the most probable state path starts with the last block, where we use the last checkpointing column to initialize recovery of the last $\sqrt{T}$ states of the most likely state path and so on, until the entire sequence decoding is reconstructed. The algorithm requires memory space proportional to $O(N\sqrt{T}+T)$ and takes computational time *O*(*TNQ*_*max*_) twice as much as conventional implementation would take because of multiple sweeps. Additional computations could be easily justified by the lower memory use, which in practice leads to substantial speedup.

Only two columns are needed for the *δ *array that stores maximum probability scores for a state at a given time-point for the algorithm to run (referring to the relationship shown in Table 2). We replace the array, needed to store the dynamic programming backtrack pointers, by a linked list. Our approximately linear memory implementation follows the observation that the backtrack paths typically converge to the most likely state path and travel all together to the beginning of the decoding table. Although the approximate linearity depends on model structure and emission sequence decoded, and is not guaranteed, this behavior is typical for the HMM topologies we use. The possibility of using *O*(*N *log(*T*)) space (in case we write to disk the most likely path before the *coalescence point*, i.e. the first state on the backtrack path where only a single candidate is left for the initial segment of the most probable state path) has only been rigorously proven for simple symmetric two-state HMM [19].

**Figure 3:**
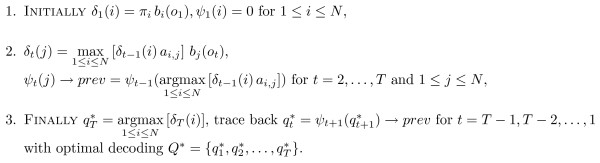
**Viterbi algorithm implementation with linked list.** Here $\psi_{t+1}(q_{t+1}^{*})\to prev$ is reference to the previous node.

The modified Viterbi algorithm is shown in Figure 3. It runs in the same *O*(*TNQ*_*max*_) time as a conventional Viterbi decoder, but takes the amount of memory *O*(*T*) as has been demonstrated by our simulations [see Section *Computational performance*].

**Figure 4:**
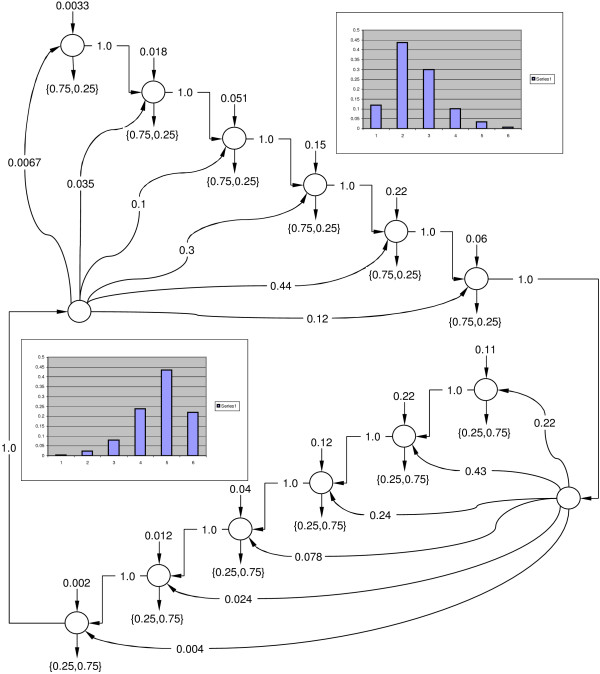
**Explicit DHMM topology.** Here the first aggregate state emits 0 with probability 0.75 and 1 with probability 0.25 and the second aggregate state emits 0 with probability 0.25 and 1 with probability 0.75. Duration histograms are shown for each aggregate state.

**Figure 5:**
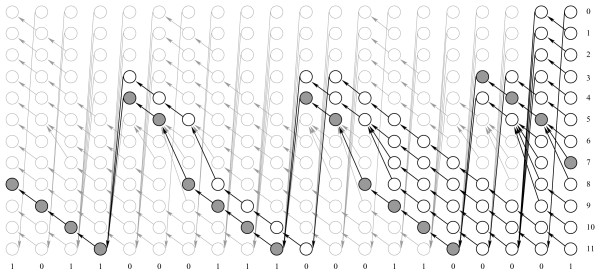
**Explicit Duration HMM trellis for the observation string shown below.** The most likely sequence of states for the observation string shown below is shaded. The lightly grayed states will be deallocated by garbage collector.

**Figure 6:**
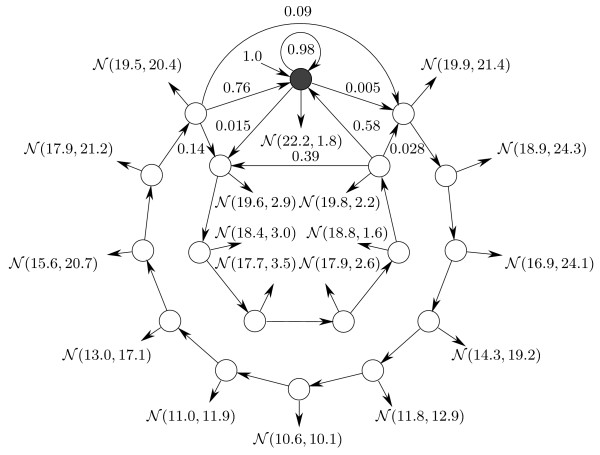
Spike detection loop topology.

**Figure 7:**
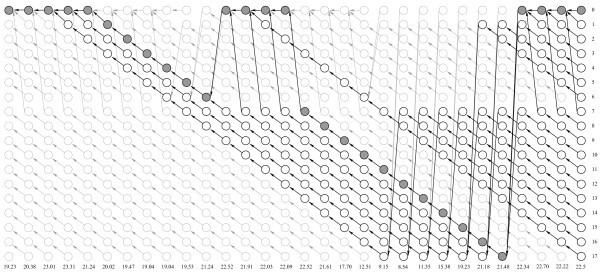
**Trellis for loopy topology used for spikes detection where shallow spike (states 1–6) and deep spike (states 7–17) are consequently decoded.** The most likely sequence of states for the sequence of observed ionic flow current blockades (in pA) shown below is shaded. The lightly grayed states will be deallocated by garbage collector.

This approach has proven to be useful in decoding the explicit Duration Hidden Markov Model (DHMM) topology introduced in [6], as can be seen in Figure 4. Although this implementation closely follows the originally proposed non-parametric duration density [20] design, the advantage is that we no longer have to use highly modified Forward-Backward and Viterbi algorithms [21]. This topology directly corresponds to the Generalized Hidden Markov Model (GHMM) used in GENSCAN [22], one of the most accurate gene structure prediction tools. The potential for a very large number of nodes in our proposed topology is compensated by introducing the linear memory Viterbi and Baum-Welch implementations with unaltered running time *O*(*STM*), where *M *is the maximum duration of an aggregate state and *S *is the number of aggregate states. An example of backtracking path compression for such a topology with discrete emissions can be seen in Figure 5, where the most likely trail of states quickly combines with all the alternative trails. Another interesting topology used by our laboratory for "spike" detection is shown in Figure 6, where the spikes are modelled as a mixture of two trajectories interconnected with an underlying set of ionic flow blockade states. The Viterbi decoding trail for this topology, detecting two sequential spikes in samples from the real continuous ionic flow blockade, is shown in Figure 7. Again, the backtracks quickly converge to the most likely state sequence.

Our particular implementation relies on the Java Garbage Collector (GC), which periodically deletes all the linked list nodes allocated in the heap that are no longer referenced by the active program stack as shaded in light gray color in Figures 5 and 7. On multiple core machines the GC could run concurrently with the main computational thread thus not obstructing execution of the method. In the lower level languages (like C/C++) "smart pointers" could be used to deallocate unused links when their reference count drops to zero, which is in some ways even more efficient than Java's garbage collection.

### Computational performance

We conducted experiments on the HMM topologies mentioned above [see Section *Viterbi decoding in linear memory*] with both artificial and real data sets, and evaluated performance of the various implementations of the Viterbi and EM learning algorithms. We describe the performance of the Java Virtual Machive (JVM) after the HotSpot Java byte code optimizer burns-in, i.e. after it optimizes both memory use and execution time within EM cycles. The linear memory, checkpointing and conventional algorithm implementations are thereby streamlined to avoid an unfair comparison due to obvious performance bottlenecks.

For the DHMM topology shown in Figure 4 we have chosen to systematically alter the size of two aggregate states from 50 to 500 when learning on an artificially generated sequence of 10,000 discrete symbols to see how the number of free learning parameters affects the performance of the EM learning algorithms. In Subfigures 8(a) – 8(c) we see that the running time of the linear implementation grows dramatically compared to conventional and checkpointing implementations, making it a very slow alternative for such a scenario. Although the linear implementation memory profile is low, as expected, for high values of *D*, the checkpointing algorithm claims the least memory. This is because the table sizes in the linear memory EM implementation grow quickly as the number of states and transitions in the model increases. Garbage collection for large *D *is the lowest for the checkpointing EM compared to other implementations.

**Figure 8:**
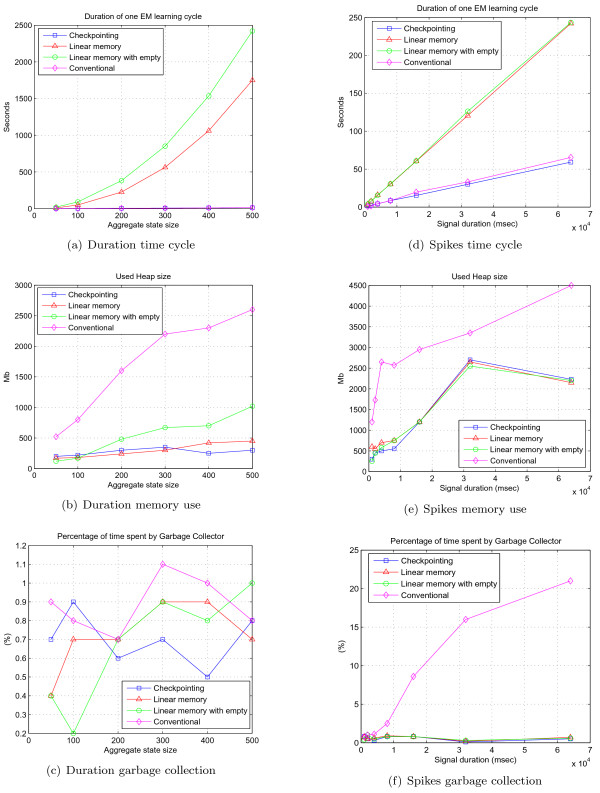
**In subfigures 8(a) – 8(c) performance of Baum-Welch used on DHMM topology with two aggregate states of various maximum duration *D*.** In subfigures 8(d) – 8(f) performance of Baum-Welch algorithm used on spike topology for various ionic flow durations is shown.

In experiments on EM learning on a spike detection HMM topology, shown in Figure 6, we have systematically varied the ionic flow duration from 1,000 ms to 64,000 ms. Although in Subfigures 8(d) – 8(f) duration of the time cycle of the linear memory implementation is not so inflated in this situation, it is still many times higher than for conventional and checkpointing algorithms. Please note that conventional and checkpointing algorithms spend almost identical time per cycle. The conventional implementation still takes the largest amount of memory and once again checkpointing takes less memory compared to the linear memory implementation in the case of long signal duration. Garbage collection in the case of the conventional implementation starts taking a substantial fraction of the CPU time for maximum signal duration, which advocates against using the conventional implementation for the analysis of long signals.

Theoretically, for the linear memory algorithm to run faster than checkpointing alternative the following condition should hold true 2*TNQ*_*max *_+ *T *(*Q *+ *E*) > *TNQ*_*max*_(*Q *+ *ED*) which reduces to the condition, unrealistic for any practical model 2 > *Q *+ *ED*. Thus, the linear memory algorithm will always run slower than checkpointing. The memory condition for the linear memory EM algorithm implementation to run in less space is 2*N*$\sqrt{T}$ > *N*(*Q *+ *ED*), which reduces to 2$\sqrt{T}$ > *Q *+ *ED *condition – quite realistic for sufficiently large values of *T*. The memory advantage is the key attribute since efforts are underway (Z. Jiang and S. Winters-Hilt) to perform segmented linear HMM processing on a distributed platform, where the speed deficiency is offset by *M*-fold speedup when using *M *nodes.

In both test scenarios shown in Figure 8 we see that conventional implementation of Baum-Welch aggressively claims very large amounts of heap space, even for modestly sized problems (in some applications, such as the JAHMM package [23], it allocates the probabilistic table *ξ *of size *N*^2 ^× *T*, although we do it in *N *× *T *through progressive summation of forward-backward tables), where both modified EM algorithm implementations have a very compact memory signature. An HMM algorithm implemented based on forward sweep strategy with silent Start/End states runs slower and takes more memory compared to the proposed backward sweep strategy in case of DHMM topology. This is because prior probabilities of the states are estimated as regular transitions from the *Start *state, thus substantially increasing *t*_*i*, *j*_(*t*, *m*) table size and time required for a recurrent step.

In Tables 5 and 6 we list the ratio of memory used by the linked list nodes referenced from the active program stack to the sequence length *T*. As could be seen, it quickly becomes proportional to 1.0 in both spike detection and the explicit DHMM topologies as the decoded sequence length grows.

**Table 5:** Memory use for Viterbi decoding on spike topology with loop sizes 6 and 11.

Ionic flow samples	Ratio of number of referenced links to sequence size
819	1.1173
10,319	1.0084
26,233	1.0042
51,233	1.0017
101,233	1.0015
151,232	1.0007

**Table 6:** Memory use for Viterbi decoding on explicit DHMM with *D *= 60 and two aggregate

Observation sequence size	Ratio of number of referenced links to sequence size
1,000	2.565
10,000	1.134
50,000	1.032
100,000	1.017
200,000	1.007

## Discussion and Conclusion

We have discussed implementation of Baum-Welch and Viterbi algorithms in linear memory. We successfully used these implementations in analysis of nanopore blockade signals with very large sample sizes (more than 3,000 ms) where the main limitation becomes processing time rather than memory overflow. We are currently working on efficient distributed implementations of the linear memory algorithms to facilitate quicker, potentially "real-time" applications.

In both of the test scenarios considered, the linear memory modification of the EM algorithm takes significantly more time to execute compared to conventional and checkpointing implementations. Despite being the fastest in many realistic scenarios, the conventional implementation of the EM learning algorithm suffers from substantial memory use. The checkpointing algorithm alleviates both of these extremes, sometimes running even faster than the conventional algorithm due to lower memory management overhead. The checkpointing algorithm seems to provide an excellent tradeoff between memory use and speed. We are trying to understand if the running time of our linear memory EM algorithm implementation can be constrained in a way similar to the checkpointing algorithm. A demo program featuring the canonical, checkpointing and linear memory implementations of the EM learning and the Viterbi decoding algorithms is available on our web site (see *Availability & requirements* section below).

## Availability & requirements

University of New Orleans bioinformatics group: 

## Authors' contributions

AC conceptualized the project, implemented and tested the Baum-Welch and Viterbi linear memory procedures. SW–H suggested focus on linear memory algorithms and outlined the idea for the Viterbi linear memory. SW–H helped with writing up the manuscript and provided many valuable suggestions throughout the study. All authors read and approved the final manuscript.

## Appendices

### Appendix A – Proofs of scaling relationships

The scaling steps in Figure 2 need additional rigorous justification. Our proofs are partially inspired by recurrences presented in [24] with further clarifications.

**Theorem 2 **$\tilde{\beta }$_*t*_(*m*) = *d*_*t *_$\bar{\beta }$_*t*_(*m*)

**Proof **Let us define $D_{t}=\frac{1}{\sum_{i=1}^{N}\beta_{t}(i)},d_{t}=\frac{1}{\sum_{i=1}^{N}\beta_{t}(i)},{\tilde{\beta }}_{t}(m)=D_{t}\beta_{t}(m)$,

$$\begin{array}{c} {\bar{\beta }}_{t}(m)=\sum_{j=1}^{N}a_{m,j}b_{j}(o_{t+1}){\tilde{\beta }}_{t+1}(j)\\ =D_{t+1}\sum_{j=1}^{N}a_{m,j}b_{j}(o_{t+1})\beta_{t+1}(j)\\ =D_{t+1}\beta_{t}(m),\\ d_{t}=\frac{1}{\sum_{i=1}^{N}{\bar{\beta }}_{t}(i)}=\frac{1}{D_{t+1}\sum_{i=1}^{N}\beta_{t}(i)},\\ {\tilde{\beta }}_{t}(m)=d_{t}{\bar{\beta }}_{t}(m)=d_{t}D_{t+1}\beta_{t}(m)=\frac{1}{D_{t+1}\sum_{i=1}^{N}\beta_{t}(i)}D_{t+1}\beta_{t}(m)=D_{t}\beta_{t}(m). \end{array}$$

Here we observe useful relationships *D*_*t *_= *d*_*t *_*D*_*t *+ 1 _and $\bar{\beta }$_*t*_(*m*) = *D*_*t *+ 1_*β*_*t*_(*m*) necessary in follow-up proves.   □

**Theorem 3 **$\tilde{T}$_*i*, *j*_(*t*, *m*) = *d*_*t *_$\bar{T}$_*i*, *j*_(*t*, *m*)

**Proof **Let us define $\tilde{T}$_*i*, *j*_(*t*, *m*) = *D*_*t*_*T*_*i*, *j*_(*t*, *m*),

$$\begin{array}{c} {\tilde{T}}_{i,j}(t,m)=d_{t}{\bar{T}}_{i,j}(t,m)\\ =d_{t}\left[{\tilde{\beta }}_{t+1}(j)a_{m,j}b_{j}(o_{t+1})\delta (i=m)+\sum_{n=1}^{N}a_{m,n}{\tilde{T}}_{i,j}(t+1,n)b_{n}(o_{t+1})\right]\\ =d_{t}\left[D_{t+1}\beta_{t+1}(j)a_{m,j}b_{j}(o_{t+1})\delta (i=m)+D_{t+1}\sum_{n=1}^{N}a_{m,n}T_{i,j}(t+1,n)b_{n}(o_{t+1})\right]\\ =d_{t}D_{t+1}\left[\beta_{t+1}(j)a_{m,j}b_{j}(o_{t+1})\delta (i=m)+\sum_{n=1}^{N}a_{m,n}T_{i,j}(t+1,n)b_{n}(o_{t+1})\right]\\ =d_{t}D_{t+1}T_{i,j}(t,m)\\ =D_{t}T_{i,j}(t,m). \end{array}$$

**Theorem 4 **$\tilde{E}$_*i*_(*γ*, *t*, *m*) = *d*_*t *_$\bar{E}$_*i*_(*γ*, *t*, *m*)

**Proof **Let us define $\tilde{E}$_*i*_(*γ*, *t*, *m*) = *D*_*t*_*E*_*i*_(*γ*, *t*, *m*),

$$\begin{array}{c} {\tilde{E}}_{i}(\gamma ,t,m)=d_{t}{\bar{E}}_{i}(\gamma ,t,m)\\ =d_{t}\left[\sum_{n=1}^{N}b_{n}(o_{t+1})a_{m,n}{\tilde{E}}_{i}(\gamma ,t+1,n)+{\bar{\beta }}_{t}(m)\text{ SCORE}(o_{t},\gamma )\delta (m=i)\right]\\ =d_{t}\left[D_{t+1}\sum_{n=1}^{N}b_{n}(o_{t+1})a_{m,n}E_{i}(\gamma ,t+1,n)+D_{t+1}\beta_{t}(m)\text{ SCORE}(o_{t},\gamma )\delta (m=i)\right]\\ =d_{t}D_{t+1}\left[\sum_{n=1}^{N}b_{n}(o_{t+1})a_{m,n}E_{i}(\gamma ,t+1,n)+\beta_{t}(m)\text{ SCORE}(o_{t},\gamma )\delta (m=i)\right]\\ =d_{t}D_{t+1}E_{i}(\gamma ,t,m)\\ =D_{t}E_{i}(\gamma ,t,m). \end{array}$$

## Supplementary Material

Additional File 1 **Supplementary materials**. Contains previously derived recurrences for linear memory HMM implementation with forward sweep and empty start/end states along with corrected recurrences. Click here for file

## References

[B1] Bilmes J (2002). What HMMs can do. Tech rep.

[B2] Rabiner L, Juang BH (1993). Fundamentals of speech recognition.

[B3] Durbin R, Eddy S, Krogh A, Mitchison G (1998). Biological sequence analysis.

[B4] Vercoutere W, Winters-Hilt S, Olsen H, Deamer D, Haussler D, Akeson M (2001). Rapid discrimination among individual DNA hairpin molecules at single-nucleotide resolution using an ion channel. Nature Biotechnology.

[B5] Vercoutere W, Winters-Hilt S, DeGuzman V, Deamer D, Ridino S, Rodgers J, Olsen H, Marziali A, Akeson M (2003). Discrimination among individual Watson-Crick base pairs at the termini of single DNA hairpin molecules. Nucleic Acids Research.

[B6] Churbanov A, Baribault C, Winters-Hilt S (2007). Duration learning for analysis of nanopore ionic current blockades. BMC Bioinformatics.

[B7] Winters-Hilt S, Landry M, Akeson M, Tanase M, Amin I, Coombs A, Morales E, Millet J, Baribault C, Sendamangalam S (2006). Cheminformatics methods for novel nanopore analysis of HIV DNA termini. BMC Bioinformatics.

[B8] Baum L, Petrie T, Soules G, Weiss N (1970). A maximization technique occurring in the statistical analysis of probabilistic functions of Markov chains. Ann Math Statist.

[B9] Hirschberg D (1975). A linear-space algorithm for computing maximal common subsequences. Communications of the ACM.

[B10] Grice J, Hughey R, Speck D (1997). Reduced space sequence alignment. CABIOS.

[B11] Tarnas C, Hughey R (1998). Reduced space hidden Markov model training. Bioinformatics.

[B12] Wheeler R, Hughey R (2000). Optimizing reduced-space sequence analysis. Bioinformatics.

[B13] Miklós I, Meyer I (2005). A linear memory algorithm for Baum-Welch training. BMC Bioinformatics.

[B14] Rabiner L (1989). A tutorial on hidden Markov models and selected applications in speach recognition. Proceedings of IEEE.

[B15] Bilmes J (1998). A gentle tutorial of the EM algorithm and its application to parameter estimation for Gaussian mixture and hidden Markov models. Tech Rep TR-97-021.

[B16] Wierstra D, Wiering M (2004). Master's Thesis.

[B17] Kingsbury N, Rayner P (1971). Digital filtering using logarithmic arithmetic. Electronics Letters.

[B18] Viterbi A (1967). Error bounds for convolutional codes and an asymptotically optimum decoding algorithm. IEEE Transactions on Information Theory.

[B19] Šrámek R, Brejová B, Vinař T (2007). On-line Viterbi algorithm and its relationship to random walks. Tech rep.

[B20] Ferguson J, Ferguson J (1980). Variable duration models for speech. Proc Symposium on the application of Hidden Markov Models to text and speech.

[B21] Mitchell C, Helzerman R, Jamieson L, Harper M (1995). A parallel implementation of a hidden Markov model with duration modeling for speech recognition. Digital Signal Processing, A Review Journal.

[B22] Burge C, Karlin S (1997). Predictions of complete gene structures in human genomic DNA. Journal of Molecular Biology.

[B23] François JM Jahmm – Java Hidden Markov Model (HMM). http://www.run.montefiore.ulg.ac.be/~francois/software/jahmm/.

[B24] Rahimi A (2000). An erratum for "A tutorial on Hidden Markov Models and selected applications in speech recognition". http://alumni.media.mit.edu/~rahimi/rabiner/rabiner-errata/rabiner-errata.html.

