# Supplementary material for: Implementing EM and Viterbi algorithms for Hidden Markov Model in linear memory
Source: BMC Bioinformatics. 2008 Apr 30;9:224. doi: 10.1186/1471-2105-9-224 (PMC2430973; doi:10.1186/1471-2105-9-224)
Supplement: Additional File 1 — Supplementary materials. Contains previously derived recurrences for linear memory HMM implementation with forward sweep and empty start/end states along with corrected recurrences. [file 1471-2105-9-224-S1.pdf]

Supplementary material

1 Previously proposed recurrences

According to notation in [1]: M is the number of states in an HMM, End is the silent end state, $Start$ is the silent start state, $T_{i,j}$ is transition probability between states i and j, $E_i(\gamma)$ is probability of emitting symbol γ from state i, $P(X)$ is probability of sequence X, x_k is the kth letter in input sequence X of length L, where X_k is the sequence of letters from the beginning of sequence X up to the sequence position k. $f(X_k, n)$ is the forward probability, i.e. is the sum of probabilities of all state paths that finish in state n at sequence position k.

$t_{i,j}(X_k, l)$ denotes the weighted sum of probabilities of state paths that finish in state l at sequence position k of sequence X and that contain at least one $i \rightarrow j$ transition, where the weight for each state path is equal to its number of $i \rightarrow j$ transitions.

$e_i(\gamma, X_k, l)$ denotes the weighted sum of probabilities of state paths that finish at sequence position k in state l for which state i reads letter γ at least once, the weight for each state path being equal to the number of times state i reads letter γ.

1.1 Transition estimate recurrences

Initialization $f(X_0, m) = \begin{cases} 1, & \text{if} \quad m = Start \\ 0, & \text{if} \quad m \neq Start \end{cases}$,

$t_{i,j}(X_0, m) = 0.$

Recurrence $f(X_{k+1}, m) = \sum_{n=1}^{M} f(X_k, n) T_{n,m} E_m(x_{k+1})$,

$$t_{i,j}(X_{k+1}, m) = \begin{cases} \sum_{n=1}^{M} t_{i,j}(X_k, n) T_{n,m} E_m(x_{k+1}), & \text{if} \quad m \neq j \\ \\ f(X_k, i) T_{i,m} E_m(x_{k+1}) + & \text{if} \quad m = j \\ \sum_{n=1}^{M} t_{i,j}(X_k, n) T_{n,m} E_m(x_{k+1}). & \end{cases} \tag{1}$$

Termination $P(X) = f(X_L, End) = \sum_{n=1}^{M} f(X_L, n) T_{n,End}$,

$$t_{i,j}(X) = t_{i,j}(X_L, End) = \begin{cases} \sum_{n=1}^{M} t_{i,j}(X_L, n) T_{n,End}, & \text{if} \quad End \neq j \\ \\ f(X_L, i) T_{i,End} + & \text{if} \quad End = j \\ \sum_{n=1}^{M} t_{i,End}(X_k, n) T_{n,End}. & \end{cases} \tag{2}$$

1.2 Emission estimate recurrences

Initialization $f(X_0, m) = \begin{cases} 1, & \text{if} \quad m = Start \\ 0, & \text{if} \quad m \neq Start \end{cases}$,

$e_i(\gamma, X_0, m) = 0$.

Recurrence $f(X_{k+1}, m) = \sum_{n=1}^{M} f(X_k, n) T_{n,m} E_m(x_{k+1})$,

$$e_i(\gamma, X_{k+1}, m) = \begin{cases} \sum_{n=1}^{M} e_i(\gamma, X_k, n) T_{n,m} E_m(x_{k+1}), & \text{if} \quad m \neq i \text{ or } x_{k+1} \neq \gamma \\ \\ f(X_k, i) T_{i,m} E_m(x_{k+1}) + & \text{if} \quad m = i \text{ or } x_{k+1} = \gamma \\ \sum_{n=1}^{M} e_i(\gamma, X_k, n) T_{n,m} E_m(x_{k+1}). & \end{cases} \tag{3}$$

Termination $P(X) = f(X_L, End) = \sum_{n=1}^{M} f(X_L, n) T_{n,End}$,

$$e_i(\gamma, X) = e_i(\gamma, X_L, End) = \sum_{n=1}^{M} e_i(\gamma, X_L, n) T_{n,End}. \tag{4}$$

2 Corrected forward sweep recurrences with empty Start and End states

In this section we provide corrected recurrences based on forward sweep strategy of the linear memory HMM implementation. Notation used here refers to the main article body.

2.1 Transition estimate recurrences

Initialization $\alpha_0(m) = \begin{cases} 1, & \text{if} \quad m = Start \\ 0, & \text{if} \quad m \neq Start \end{cases}$,

$t_{i,j}(1, m) = 0$.

Recurrence $\alpha_t(j) = \left[\sum_{i=1}^{N} \alpha_{t-1}(i) a_{i,j}\right] b_j(o_t)$ for $t = 1, 2, \ldots, T,\ i, j \neq Start, End$,

$$t_{i,j}(t, m) = \alpha_{t-1}(i)\, a_{i,m}\, b_m(o_t)\, \delta(m = j) \quad (5)$$

$$+ \sum_{n=1}^{N} t_{i,j}(t-1, n)\, a_{n,m}\, b_m(o_t). \quad (6)$$

Termination $p(O|\lambda) = \sum_{i=1}^{N} \alpha_T(i)$,

$$t_{i,j}^{END} = \sum_{m=1}^{N} t_{i,j}(T, m) a_{m,End},\ a_{i,j} = \frac{t_{i,j}^{END}}{\sum_{j \in out(S_i)} t_{i,j}^{END}},$$

where $out(S_i)$ is the set of nodes connected by edges from S_i.

2.2 Emission estimate recurrences

Initialization $\alpha_0(m) = \begin{cases} 1, & \text{if} \quad m = Start \\ 0, & \text{if} \quad m \neq Start \end{cases}$,

$e_i(\gamma, 1, m) = 0$.

Recurrence $\alpha_t(j) = \left[\sum_{i=1}^{N} \alpha_{t-1}(i) a_{i,j}\right] b_j(o_t)$ for $t = 1, 2, \ldots, T,\ i, j \neq Start, End$,

Recurrence (3) erroneously resembles formula (1) for taking forward probability $f(X_k, i)$ of coming to a state i at time point k to score the emission $E_m(x_{k+1})$ upon making a transition $T_{i,i}$, instead of adding the forward probability $f(X_{k+1}, i)$ in case $x_{k+1} = \gamma$. Here we give the correct recurrence in our notation:

$$e_i(\gamma, t, m) = \alpha_t(m)\, \delta(i = m)\, \delta(\gamma = o_t) \quad (7)$$

$$+ \sum_{n=1}^{N} e_i(\gamma, t-1, n)\, a_{n,m}\, b_m(o_t). \quad (8)$$

Termination

$$e_i^{END}(\gamma) = \sum_{m=1}^{N} e_i(\gamma, T, m) a_{m,End},\ \hat{b}_j(\gamma) = \frac{e_j^{END}(\gamma)}{\sum_{\gamma=1}^{D} e_j^{END}(\gamma)}.$$

References

[1] Miklós I, Meyer I: **A linear memory algorithm for Baum-Welch training**. *BMC Bioinformatics* 2005, **6**(231).
